# *trans*-1-Phenyl­pyrrolidine-2,5-dicarbo­nitrile

**DOI:** 10.1107/S1600536810001479

**Published:** 2010-01-16

**Authors:** Wei Han, Armin R. Ofial, Peter Mayer

**Affiliations:** a Ludwig-Maximilians-Universität, Department, Butenandtstrasse 5–13, 81377 München, Germany

## Abstract

In the title compound, C_12_H_11_N_3_, the plane of the phenyl ring and the least-squares plane of the pyrrolidine ring enclose an angle of 14.30 (6)°. The intra­cyclic N atom features a nearly trigonal-planar coordination geometry due to π-inter­actions with the aromatic system. The pyrrolidine ring is present in a twist conformation for which the closest pucker descriptor is ^C9^ *T*_C8_. Weak inter­molecular C—H⋯N and C—H⋯π contacts occur

## Related literature

For background to the synthesis, see: Han & Ofial (2009 ▶); Takahashi *et al.* (1986 ▶). For a related structure, see: Menezes *et al.* (2007 ▶). For puckering analysis, see: Cremer & Pople (1975 ▶).
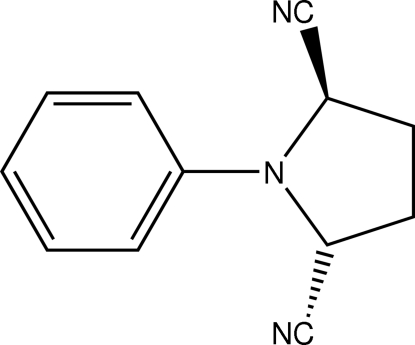


## Experimental

### 

#### Crystal data

C_12_H_11_N_3_
*M* *_r_* = 197.24
Orthorhombic, 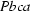

*a* = 9.1807 (1) Å
*b* = 14.5693 (2) Å
*c* = 15.7576 (2) Å
*V* = 2107.68 (5) Å^3^
*Z* = 8
Mo *K*α radiation
μ = 0.08 mm^−1^
*T* = 200 K
0.33 × 0.18 × 0.15 mm

#### Data collection

Nonius KappaCCD diffractometer
16161 measured reflections
2413 independent reflections
2109 reflections with *I* > 2σ(*I*)
*R*_int_ = 0.022

#### Refinement

*R*[*F* ^2^ > 2σ(*F* ^2^)] = 0.039
*wR*(*F* ^2^) = 0.107
*S* = 1.06
2413 reflections
136 parameters
H-atom parameters constrained
Δρ_max_ = 0.14 e Å^−3^
Δρ_min_ = −0.18 e Å^−3^

### 

Data collection: *COLLECT* (Hooft, 2004 ▶); cell refinement: *SCALEPACK* (Otwinowski & Minor, 1997 ▶); data reduction: *DENZO* (Otwinowski & Minor, 1997 ▶) and *SCALEPACK*; program(s) used to solve structure: *SIR97* (Altomare *et al.*, 1999 ▶); program(s) used to refine structure: *SHELXL97* (Sheldrick, 2008 ▶); molecular graphics: *ORTEP-3* (Farrugia, 1997 ▶); software used to prepare material for publication: *PLATON* (Spek, 2009 ▶).

## Supplementary Material

Crystal structure: contains datablocks I, global. DOI: 10.1107/S1600536810001479/fl2285sup1.cif

Structure factors: contains datablocks I. DOI: 10.1107/S1600536810001479/fl2285Isup2.hkl

Additional supplementary materials:  crystallographic information; 3D view; checkCIF report

## Figures and Tables

**Table 1:** Hydrogen-bond geometry (Å, °) *Cg* is the centroid of the C1–C6 ring.

*D*—H⋯*A*	*D*—H	H⋯*A*	*D*⋯*A*	*D*—H⋯*A*
C6—H6⋯N2^i^	0.95	2.70	3.5952 (17)	158
C8—H8*B*⋯N2^ii^	0.99	2.67	3.3777 (17)	129
C10—H10⋯N3^iii^	1.00	2.69	3.3748 (15)	126
C8—H8*A*⋯*Cg*^i^	0.99	2.74	3.6937 (13)	162
C9—H9*B*⋯*Cg*^iv^	0.99	2.88	3.5111 (13)	122

Symmetry codes: (i) 
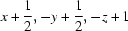
; (ii) 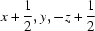; (iii) 
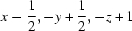
; (iv) 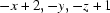.

**Fig. 1.**
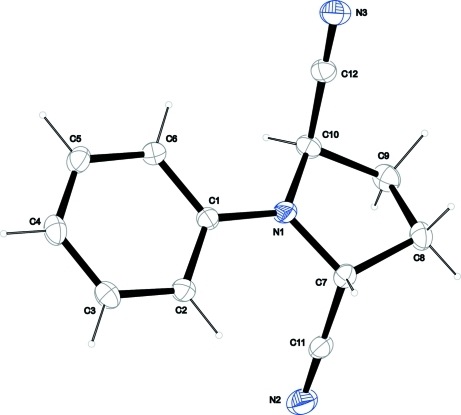
The molecular structure of the title compound, with atom labels and anisotropic displacement ellipsoids (drawn at 50% probability level) for non-H atoms.

**Fig. 2.**
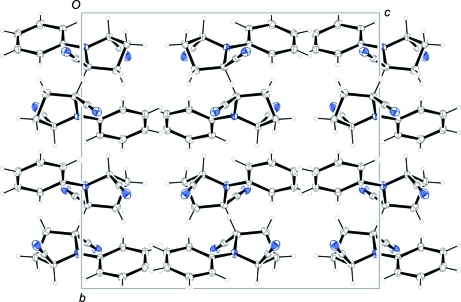
The packing of the title compound viewed along [-100].

## supplementary crystallographic information

### Comment

Under oxidative conditions, iron salts activate C(*sp*^3^)-H bonds adjacent to the nitrogen atom of tertiary amines. Hence the cross-coupling reactions of a variety of tertiary amines with cyanide were enabled in the presence of *tert*-butylhydroperoxide. Performing the reaction in the presence of 4 equivalents of trimethylsilyl cyanide allowed the twofold cyanation of *N*-phenylpyrrolidine [Han *et al.* (2009)].

The asymmetric unit of (I) contains one complete molecule which is shown in Figure 1. The plane of the phenyl ring and the least-square plane of the pyrrolidine ring enclose an angle of 14.30 (6)°, which is quite similar to the angle found in a related structure of 1,2-Dichloro-4-(pyrrolidino)-5-nitrobenzene (18.69 (14)°, Menezes *et al.* (2007)).

The intracyclic nitrogen atom is nearly in the plane defined by the three carbon atoms bonded to it. The displacement of the nitrogen atom from the plane is only 0.073 (1) Å. The planarization of the nitrogen atom may be rationalized by possible π-interaction of the lone pair with the aromatic system.

The pyrrolidine ring is present in a *twist*-conformation for which the closest pucker descriptor is ^C9^*T*_C8_ [Cremer *et al.* (1975)]. This conformation is slightly distorted towards an *envelope*-conformation *E*_C8_. In a related structure [Menezes *et al.* (2007)], the ring is twisted along the same bond, however, in the other direction (^C8^*T*_C9_).

Both carbonitrile nitrogen atoms act as acceptors in very weak contacts of the type C–H···N. Furthermore the packing features very weak C–H···π contacts with the phenyl π-system as acceptor. The packing of the title compound is shown in Figure 2.

### Experimental

Under an atmosphere of dry nitrogen, a 25 ml Schlenk flask was charged with FeCl_2_ (10 mol-/%, 13 mg). Then *N*-phenylpyrrolidine (1.0 mmol), trimethylsilyl cyanide (4.0 mmol, 0.54 ml), and MeOH (2.0 ml) were added successively by syringe. To the mixture was added dropwise *tert*-butylhydroperoxide (2.5 mmol, 0.470 ml, 5.5 *M* solution in decane) over a period of 5 minutes. The mixture was stirred at room temperature for 24 h. Subsequently, the reaction mixture was poured into a saturated aqueous NaCl solution (20 ml) and extracted with dichloromethane (three times 20 ml). The organic phases were combined, and the volatile components were evaporated in a rotary evaporator. After column chromatography on silica gel (*n*-pentane/diethyl ether = 3:1, *v*/*v*), 1-phenylpyrrolidine-2,5-dicarbonitrile was isolated as a colorless solid (54%) [Han *et al.* (2009)].

The title compound (50 mg) was dissolved in 2 ml of a mixture of dichloromethane/*n*-pentane/ethyl ether mixture (2/1/1, *v*/*v*/v). The solvent was allowed to evaporate slowly at room temperature. The thus formed crystals were suitable for X-ray analysis. mp 149–151 °C ([Takahashi *et al.* (1986)], mp 161.5–163 °C (from ethanol)).

### Refinement

All H atoms were found in difference maps. The H atoms were positioned geometrically (bond distances for phenyl-CH: 0.95 Å, for aliphatic CH: 1.00 Å, for aliphatic CH_2_: 0.99 Å) and treated as riding on their parent atoms [*U*_iso_(H) = 1.2*U*_eq_(C)].

### Crystal data

**Table:**

C_12_H_11_N_3_	*F*(000) = 832
*M**_r_* = 197.24	*D*_x_ = 1.243 (1) Mg m^−^^3^
Orthorhombic, *P**b**c**a*	Mo *K*α radiation, λ = 0.71073 Å
Hall symbol: -P 2ac 2ab	Cell parameters from 9019 reflections
*a* = 9.1807 (1) Å	θ = 3.1–27.5°
*b* = 14.5693 (2) Å	µ = 0.08 mm^−^^1^
*c* = 15.7576 (2) Å	*T* = 200 K
*V* = 2107.68 (5) Å^3^	Block, colourless
*Z* = 8	0.33 × 0.18 × 0.15 mm

### Data collection

**Table:**

Nonius KappaCCD diffractometer	2109 reflections with *I* > 2σ(*I*)
Radiation source: rotating anode	*R*_int_ = 0.022
MONTEL, graded multilayered X-ray optics	θ_max_ = 27.5°, θ_min_ = 3.4°
Detector resolution: 9 pixels mm^-1^	*h* = −11→11
CCD; rotation images; thick slices, phi/ω–scan	*k* = −18→18
16161 measured reflections	*l* = −20→20
2413 independent reflections	

### Refinement

**Table:**

Refinement on *F*^2^	Primary atom site location: structure-invariant direct methods
Least-squares matrix: full	Secondary atom site location: difference Fourier map
*R*[*F*^2^ > 2σ(*F*^2^)] = 0.039	Hydrogen site location: inferred from neighbouring sites
*wR*(*F*^2^) = 0.107	H-atom parameters constrained
*S* = 1.06	*w* = 1/[σ^2^(*F*_o_^2^) + (0.049*P*)^2^ + 0.5393*P*] where *P* = (*F*_o_^2^ + 2*F*_c_^2^)/3
2413 reflections	(Δ/σ)_max_ < 0.001
136 parameters	Δρ_max_ = 0.14 e Å^−^^3^
0 restraints	Δρ_min_ = −0.18 e Å^−^^3^

### Special details

**Table:**

Geometry. All e.s.d.'s (except the e.s.d. in the dihedral angle between two l.s. planes) are estimated using the full covariance matrix. The cell e.s.d.'s are taken into account individually in the estimation of e.s.d.'s in distances, angles and torsion angles; correlations between e.s.d.'s in cell parameters are only used when they are defined by crystal symmetry. An approximate (isotropic) treatment of cell e.s.d.'s is used for estimating e.s.d.'s involving l.s. planes.
Refinement. Refinement of *F*^2^ against ALL reflections. The weighted *R*-factor *wR* and goodness of fit *S* are based on *F*^2^, conventional *R*-factors *R* are based on *F*, with *F* set to zero for negative *F*^2^. The threshold expression of *F*^2^ > 2*σ*(*F*^2^) is used only for calculating *R*-factors(gt) *etc*. and is not relevant to the choice of reflections for refinement. *R*-factors based on *F*^2^ are statistically about twice as large as those based on *F*, and *R*-factors based on all data will be even larger.

### Fractional atomic coordinates and isotropic or equivalent isotropic displacement parameters (Å^2^)

**Table:**

	*x*	*y*	*z*	*U*_iso_*/*U*_eq_	
N1	0.91889 (10)	0.12762 (7)	0.48710 (6)	0.0339 (2)	
N2	0.63244 (13)	0.15665 (9)	0.34906 (8)	0.0515 (3)	
N3	1.27530 (11)	0.14135 (8)	0.55151 (7)	0.0460 (3)	
C1	0.84192 (11)	0.11277 (7)	0.56211 (6)	0.0289 (2)	
C2	0.71584 (12)	0.05831 (8)	0.56245 (7)	0.0331 (3)	
H2	0.6806	0.0329	0.5109	0.040*	
C3	0.64254 (13)	0.04149 (8)	0.63784 (8)	0.0395 (3)	
H3	0.5569	0.0049	0.6373	0.047*	
C4	0.69229 (13)	0.07717 (9)	0.71358 (8)	0.0414 (3)	
H4	0.6423	0.0647	0.7651	0.050*	
C5	0.81566 (13)	0.13119 (9)	0.71352 (7)	0.0393 (3)	
H5	0.8501	0.1561	0.7655	0.047*	
C6	0.89031 (12)	0.14984 (8)	0.63898 (7)	0.0338 (3)	
H6	0.9744	0.1878	0.6401	0.041*	
C7	0.87833 (12)	0.08771 (7)	0.40605 (6)	0.0313 (2)	
H7	0.8707	0.0195	0.4112	0.038*	
C8	1.00567 (13)	0.11327 (8)	0.34813 (7)	0.0385 (3)	
H8A	1.0857	0.0680	0.3527	0.046*	
H8B	0.9742	0.1176	0.2882	0.046*	
C9	1.05183 (14)	0.20663 (8)	0.38243 (8)	0.0405 (3)	
H9A	0.9888	0.2561	0.3598	0.049*	
H9B	1.1544	0.2202	0.3676	0.049*	
C10	1.03301 (11)	0.19671 (7)	0.47859 (7)	0.0326 (3)	
H10	1.0003	0.2562	0.5038	0.039*	
C11	0.73934 (13)	0.12635 (8)	0.37363 (7)	0.0349 (3)	
C12	1.16995 (12)	0.16549 (7)	0.51982 (7)	0.0342 (3)	

### Atomic displacement parameters (Å^2^)

**Table:**

	*U*^11^	*U*^22^	*U*^33^	*U*^12^	*U*^13^	*U*^23^
N1	0.0323 (5)	0.0433 (5)	0.0262 (5)	−0.0116 (4)	0.0000 (4)	0.0001 (4)
N2	0.0473 (7)	0.0588 (7)	0.0483 (6)	0.0060 (5)	−0.0134 (5)	−0.0106 (5)
N3	0.0382 (6)	0.0456 (6)	0.0542 (7)	−0.0001 (5)	−0.0083 (5)	−0.0055 (5)
C1	0.0275 (5)	0.0320 (5)	0.0270 (5)	0.0013 (4)	−0.0005 (4)	0.0025 (4)
C2	0.0315 (6)	0.0356 (6)	0.0321 (5)	−0.0039 (4)	0.0000 (4)	−0.0001 (4)
C3	0.0342 (6)	0.0412 (6)	0.0432 (6)	−0.0030 (5)	0.0068 (5)	0.0056 (5)
C4	0.0421 (7)	0.0493 (7)	0.0327 (6)	0.0067 (5)	0.0094 (5)	0.0067 (5)
C5	0.0426 (7)	0.0476 (6)	0.0277 (5)	0.0067 (5)	−0.0022 (5)	−0.0020 (5)
C6	0.0311 (6)	0.0391 (6)	0.0311 (6)	0.0004 (4)	−0.0027 (4)	−0.0014 (4)
C7	0.0351 (6)	0.0322 (5)	0.0267 (5)	−0.0007 (4)	0.0003 (4)	0.0000 (4)
C8	0.0388 (6)	0.0453 (7)	0.0315 (6)	0.0038 (5)	0.0070 (5)	0.0045 (5)
C9	0.0384 (6)	0.0427 (6)	0.0405 (6)	−0.0041 (5)	0.0031 (5)	0.0128 (5)
C10	0.0290 (5)	0.0310 (5)	0.0379 (6)	−0.0032 (4)	0.0005 (4)	0.0036 (4)
C11	0.0387 (6)	0.0369 (6)	0.0289 (5)	−0.0030 (5)	−0.0031 (5)	−0.0057 (4)
C12	0.0328 (6)	0.0312 (5)	0.0388 (6)	−0.0052 (4)	0.0003 (5)	−0.0022 (4)

### Geometric parameters (Å, °)

**Table:**

N1—C1	1.3939 (13)	C5—H5	0.9500
N1—C7	1.4519 (14)	C6—H6	0.9500
N1—C10	1.4591 (13)	C7—C11	1.4853 (16)
N2—C11	1.1437 (16)	C7—C8	1.5291 (15)
N3—C12	1.1438 (16)	C7—H7	1.0000
C1—C6	1.3987 (15)	C8—C9	1.5238 (17)
C1—C2	1.4034 (15)	C8—H8A	0.9900
C2—C3	1.3873 (15)	C8—H8B	0.9900
C2—H2	0.9500	C9—C10	1.5320 (16)
C3—C4	1.3796 (18)	C9—H9A	0.9900
C3—H3	0.9500	C9—H9B	0.9900
C4—C5	1.3791 (18)	C10—C12	1.4864 (15)
C4—H4	0.9500	C10—H10	1.0000
C5—C6	1.3868 (16)		
			
C1—N1—C7	123.63 (9)	N1—C7—H7	110.2
C1—N1—C10	123.29 (9)	C11—C7—H7	110.2
C7—N1—C10	112.31 (8)	C8—C7—H7	110.2
N1—C1—C6	120.89 (10)	C9—C8—C7	102.62 (9)
N1—C1—C2	120.60 (9)	C9—C8—H8A	111.2
C6—C1—C2	118.49 (10)	C7—C8—H8A	111.2
C3—C2—C1	120.22 (10)	C9—C8—H8B	111.2
C3—C2—H2	119.9	C7—C8—H8B	111.2
C1—C2—H2	119.9	H8A—C8—H8B	109.2
C4—C3—C2	120.91 (11)	C8—C9—C10	103.60 (9)
C4—C3—H3	119.5	C8—C9—H9A	111.0
C2—C3—H3	119.5	C10—C9—H9A	111.0
C5—C4—C3	119.10 (11)	C8—C9—H9B	111.0
C5—C4—H4	120.5	C10—C9—H9B	111.0
C3—C4—H4	120.5	H9A—C9—H9B	109.0
C4—C5—C6	121.22 (11)	N1—C10—C12	110.86 (9)
C4—C5—H5	119.4	N1—C10—C9	103.71 (9)
C6—C5—H5	119.4	C12—C10—C9	111.46 (9)
C5—C6—C1	120.06 (11)	N1—C10—H10	110.2
C5—C6—H6	120.0	C12—C10—H10	110.2
C1—C6—H6	120.0	C9—C10—H10	110.2
N1—C7—C11	111.78 (9)	N2—C11—C7	179.49 (13)
N1—C7—C8	103.38 (9)	N3—C12—C10	179.91 (15)
C11—C7—C8	111.05 (9)		
			
C7—N1—C1—C6	177.08 (10)	C10—N1—C7—C11	−102.29 (11)
C10—N1—C1—C6	−13.76 (16)	C1—N1—C7—C8	−172.55 (10)
C7—N1—C1—C2	−1.36 (16)	C10—N1—C7—C8	17.22 (12)
C10—N1—C1—C2	167.81 (10)	N1—C7—C8—C9	−33.54 (11)
N1—C1—C2—C3	177.94 (10)	C11—C7—C8—C9	86.48 (11)
C6—C1—C2—C3	−0.53 (16)	C7—C8—C9—C10	37.66 (11)
C1—C2—C3—C4	−0.45 (18)	C1—N1—C10—C12	76.32 (13)
C2—C3—C4—C5	0.83 (18)	C7—N1—C10—C12	−113.42 (10)
C3—C4—C5—C6	−0.22 (18)	C1—N1—C10—C9	−163.96 (10)
C4—C5—C6—C1	−0.78 (18)	C7—N1—C10—C9	6.30 (12)
N1—C1—C6—C5	−177.33 (10)	C8—C9—C10—N1	−27.35 (12)
C2—C1—C6—C5	1.14 (16)	C8—C9—C10—C12	91.97 (11)
C1—N1—C7—C11	67.93 (13)		

### Hydrogen-bond geometry (Å, °)

**Table:**

Cg is the centroid of the C1–C6 ring.

**Table:**

*D*—H···*A*	*D*—H	H···*A*	*D*···*A*	*D*—H···*A*
C6—H6···N2^i^	0.95	2.70	3.5952 (17)	158
C8—H8B···N2^ii^	0.99	2.67	3.3777 (17)	129
C10—H10···N3^iii^	1.00	2.69	3.3748 (15)	126
C8—H8A···Cg^i^	0.99	2.74	3.6937 (13)	162
C9—H9B···Cg^iv^	0.99	2.88	3.5111 (13)	122

Symmetry codes: (i) *x*+1/2, −*y*+1/2, −*z*+1; (ii) *x*+1/2, *y*, −*z*+1/2; (iii) *x*−1/2, −*y*+1/2, −*z*+1; (iv) −*x*+2, −*y*, −*z*+1.
